# Modelling carbon emissions of diesel trucks on longitudinal slope sections in China

**DOI:** 10.1371/journal.pone.0234789

**Published:** 2020-06-16

**Authors:** Yaping Dong, Jinliang Xu, Chenwei Gu

**Affiliations:** School of Highway, Chang'an University, Xi'an, China; Tongii University, CHINA

## Abstract

Carbon emissions are the primary reason that contributes to global warming. The gradient has a significant impact on the carbon dioxide (CO_2_) emissions produced by trucks. The aim of the current paper is to propose a carbon emission quantification model for diesel trucks on longitudinal slope sections and investigate the influence of gradient on the carbon emissions of trucks for use in the low-carbon highway design. The law of conservation of mechanical energy, the first law of thermodynamics, and the vehicle longitudinal dynamics theory were adopted for deriving the carbon emission model of the trucks on the flat, uphill, downhill and round-trip longitudinal slope segments. Three kinds of common trucks were chosen to conduct the field test. Following the test data, the model demonstrates a high accuracy. The minimum gradient which is expected to impact carbon emissions of trucks on the round-trip longitudinal slope sections was the balance gradient as revealed. The gradient of the longitudinal slope is required to be avoided to be greater in comparison with the balance gradient for the achievement of the two-way traffic low carbon operation on a highway. The results of this study are valuable to researchers interested in low carbon road design and low carbon transportation control.

## Introduction

Vehicles show high CO_2_ emissions on longitudinal slopes [1–4]. Trucks take a large proportion of all transportation in cargo terminals [5], such as that in Shaanxi Province (about 39.98%) [6]. Heavier loads cause trucks to generate more carbon emissions than passenger cars [7]. The study of carbon emissions from trucks has a more practical impact on these roads compared to passenger cars. The forecasting of CO_2_ emissions of trucks on longitudinal slopes is important for low-carbon high design and low-carbon traffic operation and has attracted significant attention. Relevant research has shed light on the fact that gradient is an important factor affecting carbon emissions of trucks [1–6].

Research on environmentally friendly transportation was focused on improving fuel economy and reducing vehicle emissions [3,4]. Most scholars focused on the study of driving behavior. They focused on various micro carbon emission models, including the Comprehensive Modal Emissions Model (CMEM) [4], Virginia Tech Comprehensive Power-based Fuel consumption Model (VT-CPFM) [8] and Motor Vehicle Emission Simulator (MOVES) [9] designed to reflect the real-time operating conditions of vehicles. The micro carbon emission models were established by using a large amount of measured data in laboratory dynamometer tests [3,4]. Ko et al. [2] extracted the second-by-second speed, vehicle specific power and carbon emissions data of vehicles on uphill sections by using the MOVES. They indicated that controlling the gradient and the speed can minimize adverse environmental effects from vehicle movements.

The different gradients of the longitudinal slope determine that there are three types of longitudinal slope on a vertical profile, namely flat straight road, uphill, and downhill [3]. Fuel consumption and CO_2_ emissions are closely correlated with traction force and running resistance [3,10,11]. Vehicle fuel consumption models based on vehicle dynamics theory have a high level of competence. Most scholars are dedicated to exploring the fuel consumption of vehicles under a certain driving force or vehicle power loads [4,10–12] using the vehicle dynamics theory. Barth and Scora et al. [4] indicated that the driving power of vehicle on uphill sections is influenced by factors such as engine transmission coefficient, instantaneous speed, road gradient, frontal vehicle area, and road friction coefficient. Kang et al. [11] suggested that fuel consumption is approximately proportional to the total propulsion, together with the introduction of a fuel consumption model for a vehicle operating at cruising speed. The formula of the total propulsive work performed by the vehicle in the fuel consumption model has been adapted from the propulsive work model proposed by Chang and Morlok [10]. It has numerous explanatory variables, including road gradient, speed, the front area of the vehicle, pavement roughness. Mechanical resistance, engine efficiency, transmission efficiency, and wind speed were not taken into consideration while calculating the propulsive force of the vehicle, thereby reducing the accuracy of the fuel consumption forecast model. Demir et al. [3] assert that different driving behaviors have been adopted on the uphill and downhill segments has resulted in different traction and fuel consumption on those segments. When the vehicle travels uphill along a straight trajectory, the tractive effort of the vehicle produced by the engine is designed to overcome external resistance. While going downhill, the vehicle's gravity potential energy has ability to overcome the work performed by running resistance. Meng and Xu [13] proposed that brakes are going to dissipate the overall mechanical energy of trucks on a longitudinal slope with a large gradient. Varying driving behaviors on the uphill and downhill segments have been adopted, resulting in different traction forces on those segments [10,11]. It should be noted that the gradient of the longitudinal slopes has a significant impact on the propulsive energy of vehicles and that the fuel consumption and carbon emissions of vehicles on the uphill and downhill segments do not theoretically show complete consistency.

Previous research has focused on quantifying fuel consumption and CO_2_ emissions on uphill segments and has shown that vehicle fuel consumption on uphill segments augments linearly as the slope increases [1–3]. Few studies have been devoted to exploring the CO_2_ emission rules of vehicles on downhill roads. Jia et al. [14] obtained instantaneous speed and fuel consumption data based on the operating conditions of a truck traversing longitudinal slope of the expressways in China. The assumption that the carbon emission rules of vehicles on uphill and downhill are consistent, followed by the consideration of both the CO_2_ emissions of vehicles on uphill and downhill segments as a quantitative model that reduces the accuracy of the model [10,15]. Chang and Morlok [10] established a regression model between the constant speed and fuel consumption of vehicle on downhill while braking is inexistent on downhill sections, and argued that different types of vehicle loads have different balance gradients on downhill sections. The balance gradient refers to the gradient in the case when the vehicle requires no propulsive force and no braking for the maintenance of a constant speed on the downgrade. It should be noted that the divided cases of the driving force on the downhill have an important role in exploring the carbon emissions rules of vehicles on downhill sections. Demir et al. [3] suggested that the fuel consumption rule on the downhill section is not always the same as the gradient increases. When the vehicle slides downhill by gravity potential without traction effort, the fuel consumption rate refers to the idle fuel rate, and the fuel consumption is inclined to showing stability.

Highways are generally constituted by integral subgrade. The round trip of the route is symmetrical. For the governments and road operation management agencies, the control of the total carbon emissions of the two-way traffic flow on a highway constitutes a direct means of attaining the low-carbon highway operations. Associated research indicated that, in comparison with the flat straight sections, vehicles require more driving energy while going uphill, together with saving the driving energy while going downhill [10,15]. Nonetheless, there are few studies that have dedicated to investigating that, subjected to what circumstances, the vehicle's CO_2_ emissions on the round-trip longitudinal slope sections are equivalent to that on flat sections. Through a real-world experiment carried out by Boriboonsomsin and Barth [15], it was confirmed that the gradient has a significant influence on the fuel economy of two-way traffic, that is, by controlling the road gradient within a certain range, the round-trip longitudinal slope can achieve the same fuel consumption rate as the flat straight section. The test passenger cars were required for the maintenance of a constant speed of 96km/h in the field test. The correlation appears to be linear within the range of grades between -2% and 2%, suggesting that there is expected to be no difference in fuel consumption among flat route and the round-trip hilly route within this gradient range. In general, the overall fuel economy of the plain route is deemed as better as compared with the mountainous round-trip route. Downhill sections have the lowest fuel consumption, followed by flat sections, and then by uphill sections. Nonetheless, under what scenarios, are the issue of the extra fuel consumed by vehicles on the uphill section offsetting the fuel saved on the downhill section as compared with the flat sections are unclear. Owing to lack of theoretical basis and the insufficient measured data in the scenarios with various gradients, the balance gradient with the same fuel consumption for a flat route and a round-trip hilly route is quite difficult to define accurately. It is deemed as not possible to indicate a credible balance gradient recommendation for the low-carbon two-way traffic on the longitudinal slope. Kang et al. [11] suggested that no propulsive force is likely to be essential for driving on downgrade sections with a large gradient. A negative slope resistance acts on the vehicle and it nullifies the impact of other resistances. The road segment where no propulsive force is necessary, is dependent on the steepness of the downgrade section. Following energy conservation [13], it is evident that, with the vehicle descending at idle speed on the downhill with a large gradient, the braking behavior is expected to consume the mechanical energy of the vehicle [13]. It is worth attaching the significance that some of the additional fuel consumption anticipated during uphill travel is likely to be avoided through the avoidance of the braking behavior on the downhill by controlling the gradient.

Previous researchers have revealed the influence of gradient on carbon emissions, mainly focusing on the relationship between vehicle power loads and fuel consumption based on vehicle dynamics theory [4,10,11], and building a carbon emission model from the microscopic perspective encompassing vehicle operating conditions [2,4,9]. The carbon emission data measured by the laboratory dynamometer are unable to reflect on the vehicle's operation on actual roads. Moreover, the default parameters in the models, for instance, vehicle type, fuel characteristics, and weather condition, are not in line with the current status in China, and cannot be directly put to use to predict the carbon emissions of the vehicle on the longitudinal slope sections in China [7,16]. Relevant research on vehicle fuel consumption under certain driving force or vehicle power loads based on vehicle dynamics theory [4,10,11] provides a theoretical basis for establishing a mathematical model of truck's carbon emission on longitudinal slopes in China.

The current study was carried out for not just investigating the carbon emission quantification model of trucks on longitudinal slope sections suitable for China, but also clarifying the minimum gradient impacting truck's carbon emissions on the round-trip of the longitudinal slope. The vehicle longitudinal dynamics theory [17], the law of conservation of mechanical energy [13] and the first law of thermodynamics [18] are employed to primarily establish the carbon emission model of trucks on the uphill, downhill and round trip longitudinal slope segments, and the factors like the vehicle performance and fuel characteristic in China were taken into account. To explore the influence of gradient on carbon emissions of two-way traffic flow, the speed was required to be uniform to avoid the interference of speed fluctuation on carbon emissions. The reliability of the conclusions is confirmed by the results of field tests. This investigation has the potential to put forward specific gradient recommendations for the low-carbon longitudinal slope designs. The model, established by the theoretical analysis and empirical research, can enhance the reliability of the results.

The rest of the paper is organized as hereunder. In Section 2, the conversion between energy, diesel consumption and CO_2_ emission are clarified. The CO_2_ emission model of diesel trucks on longitudinal slope segments in China are established. Moreover, the selection of different route typologies and a consistent number of drivers in field trials provides reliable data for verifying the theoretical model, and gives value to the work. In Section 3, the results obtained from the analysis of the collected field data are presented and discussed in detail, and the accuracy of the model is confirmed by the field-tested fuel consumption data. Moreover, the minimum gradient value, impacting the truck's CO_2_ emissions on longitudinal slopes is clarified. Section 4 discusses the entire investigation, together with highlighting the limitations of the work and prospective research directions. Eventually, the key findings of the proposed study are summarized in Section 5.

## Methods

### Conversion between energy, diesel consumption, and CO_2_ emissions

Gasoline combustion releases heat, whereas the engine converts the combustion heat into mechanical energy [17,18]. The theory of vehicle internal combustion engine power [18] figures out a methodology for the estimation of fuel consumption in accordance with the energy from fuel combustion in the engine. Considering the combustion heat, transmission efficiency and fuel utilization rate of different types of fuels, the correlation existing between the propulsive energy and fuel consumption of trucks could be attained [3,18,19], as demonstrated in Eqs (1) and (2).

$$Q=Q_{e}+Q_{0},$$ (1)

$$Q=\frac{10^{-3}W_{e}}{n_{tf}n_{i}dA}{+q}_{0}t,$$ (2)

where Q, Q_0_ and Q_e_ indicate the total fuel consumption(L), the fuel consumption at idle state, the fuel consumption caused by propulsive energy, respectively. q_0_ is a representation of the fuel consumption rate at the idle state (L/s). Vehicles need stable power in idle state to maintain vehicle speed and provide the normal operation of all parts of the vehicle, such as air conditioning, generator, power steering pumps, and air pumps. t refers to the travel time (s). W_e_ indicates the output energy of the truck engine (J). n_tf_ represents the transmission efficiency (%). While predicting truck power performance, transmission efficiency is typically considered to be 85% [17,18]. n_i_ indicates the fuel utilization rate of engine (%). A refers to the average low calorific value of diesel (TJ/Gg). d denotes the diesel density (kg/L).

The Intergovernmental Panel on Climate Change (IPCC) [20] provides a standardized and authority methodology for the carbon emissions accounting. It specifies the conversion of fuel consumption and carbon emissions, as demonstrated in Eq (3). The use of IPCC accounting methodology makes the research results of this investigation comparable to the existing research results in other countries [7,16].

$$C=ABJKdQ\times 10^{-3},$$ (3)

where, C represents the carbon emissions (kg). J indicates the potential carbon emission factor of fuel (t/TJ). B denotes the carbon oxidation rate (%). K represents the carbon conversion coefficient.

Diesel vehicles are required to use urea additive as an exhaust gas purifying agent to purify exhaust gas by China's National Motor Vehicle Emission Standard [21]. The formula proposed by the IPCC to calculate the carbon dioxide generated in this tail gas purification process is as follows:

$$C_{u}=0.733MP,$$ (4)

where, C_u_ represents the carbon emissions generated during tail gas purification (kg). M is the urea additive (kg). The amount of urea additive used is usually obtained based on the ratio of diesel truck fuel and urea consumed. P is the urea concentration (%).

In recent years, the performance of China's diesel trucks have been required to meet the National Fifth-phase Motor Vehicle Pollutant Emission standards [21]. Diesel trucks are generally equipped with compression ignition engines. Small and medium diesel trucks are generally equipped with a common compression-ignition engine. Heavy-duty diesel trucks and Tractors are usually equipped with a Xichai compression-ignition engine [22]. Moreover, the characteristic of diesel supplied to diesel trucks must meet China's National Fifth-phase Standards of Diesel for Motor Vehicles [23], and the recommended energy values specified in "National Communication on Climate Change of China" [22, 24, 25]. According to the standards and statistics of Chinese truck performance and fuel characteristics, relevant data on Chinese truck performance and diesel characteristics can be obtained. For common compression-ignition engines usually used in light vehicles, the fuel utilization rate is 40%. For the Xichai compression-ignition engine generally used in heavy vehicles and Tractors, the fuel utilization rate is 45% [12,26]. For the diesel currently puts to use in Chinese trucks, the average low calorific value is 43.330 (TJ/Gg), the potential carbon emission factor is 20.2 (t/TJ), the carbon oxidation rate is 99%, the carbon conversion coefficient is 44/12. The density of diesel type 0# and -10# is 0.835 kg/L and 0.84 kg/L, respectively [20]. The concentration of urea additive currently supplied for diesel trucks in China is 35% [24].

Using the vehicle's internal combustion engine power theory and IPCC accounting method, and considering the exhaust gas purifying agent added for diesel trucks, the carbon emissions of diesel supplied for trucks in China could be calculated in accordance with Eqs (5) and (6).

$${CO}_{2(0\#)}=2.135W_{i}\times 10^{-7}+2.626q_{0}t+3.030PW_{i}\times 10^{-8}+0.373Pq_{0}t,$$ (5)

$${CO}_{2(-10\#)}=2.135W_{i}\times 10^{-7}+2.642q_{0}t+2.980PW_{i}\times 10^{-8}+0.368Pq_{0}t,$$ (6)

where, CO_2(0#)_ and CO_2(-10#)_ represent the carbon emissions (kg) of diesel with a density of 0# and -10#, respectively.

### CO_2_ emission model

This section is conducted to establish the carbon emissions quantitative model of trucks on different gradients of the longitudinal slope, i.e., flat straight road, uphill road, downhill, and round-trip longitudinal slope combination sections. Then, the carbon emissions of trucks on the flat road section and round-trip longitudinal slope combination section are compared to reveal the influence of gradient on carbon emissions.

#### Flat road and uphill

The energy of a truck, in the course of its operations, primarily comprises the kinetic energy, gravitational potential energy, thermal energy, and the chemical energy of the fuel [18]. Based on the law of conservation of energy, in the case of the existence of no external interference, the total energy of the complete truck system remains comparatively more stable, and the energy is mutually converted as well [13]. Thermal energy refers to the portion of the energy, generated through the combustion of fuel in the engine, dissipated by the different kinds of truck component friction and running resistance. The key causes that result in the energy loss during the truck driving primarily include engine fuel utilization, transmission system efficiency, rolling resistance, air resistance, and braking friction [13,17–19].

On level terrain, trucks need propulsive force to overcome rolling resistance and air resistance to maintain cruising speed. Additional propulsive force is required for driving on upgrade sections because of the grade impact. As several previous investigations revealed [10,11], the truck propulsive energy supplied by engine needed to overcome the total resistances on the flat road section and the uphill section is demonstrated in Eq (7). When the truck is traveling on a flat road, it is not necessary to overcome the potential energy of gravity, and the gradient is zero. The gravitational potential energy is demonstrated in Eq (8).

$$W_{e}=W_{r}+W_{a}+E_{g},$$ (7)

$$E_{g}=mgSi,$$ (8)

where W_e_, W_r_, and W_a_ refer to the work (N·m) performed by driving force, rolling resistance, and air resistance, correspondingly. E_g_ indicates the gravitational potential energy (N·m). m stands for the truck mass (kg). *i* represents the gradient (%).Also, g is the gravitational acceleration, which is generally 9.8m/s. And, S refers to the travel distance (m).

The engine burns the fuel, together with providing an effective driving force for the truck with the help of the transmission system, as demonstrated in Eq (9). Transmission efficiency refers to the ratio of effective power to indicated power, which is reflected in the mechanical losses of the different kinds of components of the transmission (e.g., gearbox, main reducer, clutch, universal joint, axle and wheel). The energy transfer of the transmission system is demonstrated in Eq (10) [18].

$$F_{e}=F-F_{t},$$ (9)

$$W_{e}=n_{tf}W=W-W_{t},$$ (10)

where, F denotes the indicated driving force (N). F_e_ represents the effective driving force (N), which is numerically equal to the total resistance of the truck. F_t_ is an indication of the resistance (N) from the transmission system. W indicates the energy output by the engine (J). W_t_ refers to the energy loss (J) caused by the engine to maintain the normal operation of the transmission system.

The empirical calculations for the air resistance and rolling resistance are demonstrated in Eqs (11) and (12). The basic resistance formulas are adapted from relevant research [13,17,27, 28].

$$F_{a}=\frac{1}{2}AC_{D}\rho {\left(v+v^{\prime }cos\theta \right)}^{2},$$ (11)

$$F_{r}=mgC_{r}(3.6C_{2}v+C_{3})\times 10^{-3}\cos i,$$ (12)

where v and v' refer to the truck speed and wind speed (m/s), respectively. θ denotes the angle between the wind direction and the driving direction. A refers to the frontal area of the vehicle (m^2^), which is the area of the car from the front to the rear. ρ implies the air density, which is, in general, 1.2258 N·s^2^/m^4^. The air resistance coefficient C_D_ is figured out by the shape of the truck. The shape of the front, rear, and bottom of the truck, wheels and wheel wells, window, rearview mirrors, and mudguards all significantly impact the air resistance coefficient. The air resistance coefficients of the different types of truck shapes could be attained from the associated documents [27]. The rolling factor C_r_ is associated with the actual road pavement type as well as the pavement condition, typical values are available in the literature [28]. The C_2_ and C_3_ parameters correlate with the tire type, and the parameter values are determined empirically. For the mixed tires generally utilized in the trucks in China currently, C_2_ = 6, C_3_ = 0.068 [17].

To ensure the comparability between the carbon emission data of vehicles on the longitudinal slopes with different gradients and lengths, the carbon emission is expressed as the carbon emission rate of the vehicle for 100 kilometers. The carbon emission rate on the flat road and uphill sections are determined with the energy outputted from the engine, as shown in Eq (13).

$$C=C_{e}+C_{0},$$ (13)

where C_e_ indicates the carbon emission rate (kg/100km), which is determined with the energy produced by the engine. C_0_ refers to the carbon emission rate at the idle state (kg/100km).

#### Downhill

All through the downhill process of trucks, the partial gravity parallel to the direction of the road surface does positive work, whereas the rolling resistance, air resistance and braking force perform negative work. There are three scenarios of driving behavior on downhill sections that are subjected to different gradient conditions.

In scenario I, the gradient is slower, coupled with the smaller gravitational potential energy. The driver requires taking a refueling behavior to provide the driving force for trucks to maintain a cruising speed. The energy relationship is demonstrated in Eq (14).

$$W_{e}=W_{r}+W_{a}-E_{g},$$ (14)

The carbon emission rate in this scenario is determined by the propulsive energy produced by the engine, as illustrated in Eq (16).

The downhill segment with no propulsive force as essential, has a dependence on the steepness of the downgrade section [11] and the gravity of the vehicle. In scenario II, the gradient gradually increases; the gravitational potential energy becomes larger. Gravitational potential energy could offset the work performed by the air resistance and rolling resistance, as shown in Eq (15). The gradients in this scenario referred to the critical gradient, and could be attained. Trucks primarily rely on the inertia to maintain the cruising speed. The accelerator pedal is usually depressed lightly to avoid the engine reverse traction resistance [18]. The carbon emission rate in this scenario is basically equals to that at idle state.

$$E_{g}=W_{r}+W_{a},$$ (15)

The transmission efficiency of the transmission system is correlated with the direction of the torque flow. As the truck accelerates, the torque flow direction is transmitted from the engine to wheels. When the truck travels downhill in the gear position without refueling, and the gravity is greater than the driving resistance, the torque flow direction of the transmission system is reversed. The truck was traveled under the condition of a motored engine. The energy from wheel rotation drives the operation of the transmission system [18]. In scenario III, the truck maintains a cruise speed, and slides downhill in the gear position without refueling. The transmission of torque is shown in Eqs (16)–(18) [18].

$$W^{\prime }=n_{tf}^{\prime }W,$$ (16)

$$n_{tf}^{\prime }=n_{tf}/n_{w},$$ (17)

$$W_{t}^{\prime }=(1-n_{tf}^{\prime })W,$$ (18)

where W' indicates the energy transmitted from wheels to the engine (J). n_tf_' refers to the reverse transmission efficiency. n_w_ suggests the transmission efficiency of the wheel, which is typically considered to be 0.985 [17,18]. W_t_' represents the energy loss (J) in the transmission system under the condition of a motored engine.

The reverse traction of the transmission system consumes a portion of the truck's mechanical energy. A part of the gravitational potential energy offsets the energy consumed by the reverse traction of the transmission system. The rest of gravitational potential energy offsets the negative work carried out by the rolling resistance and air resistance [18]. The truck's energy relationship in this scenario is demonstrated in Eq (19).

$$E_{g}=W_{r}+W_{a}+W_{t}^{\prime },$$ (19)

The gradient of the truck maintains the cruise speed, i_B_, which could be calculated based on Eq (28), termed as the balance gradient. In scenario III, the carbon emission rate is equals to that at idle state.

In scenario IV, the gradient is large, part of the reduced gravitational potential energy offsets the negative work performed by the driving resistance, whereas the other part is converted into the kinetic energy. Brake pedal needs to be stepped on to maintain a cruising speed, and the energy conversion correlation of the truck is demonstrated in Eq (20).

$$E_{g}=W_{r}+W_{a}+W_{t}^{\prime }+W_{h},$$ (20)

where W_h_ refers to energy loss (J), which is caused by the brake.

The brakes used in the downhill segments are not conducive to the maintenance of the truck's mechanical energy. The braking force required for the truck in scenario IV, coupled with the resulting energy loss, could be attained from Eq (20). With the deceleration of the truck, the engine is in a forced idle state, and the carbon emission rate equals to the idle carbon emission rate [3].

When the gradient is less than the critical gradient, the driver needs to step on the accelerator pedal to maintain the vehicle speed. When the gradient is greater than or equal to the critical gradient, the driver needs to step on the accelerator pedal gently to avoid reverse drag force from the engine. There is no obvious difference in driving behavior among these scenarios, which makes it impossible to measure the critical slope value in actual road conditions accurately. In scenario III, the driving behavior of the vehicle is to release the accelerator pedal and to slide downhill in the gear position. When the gradient exceeds the balance gradient, the brake pedal needs to be depressed to control the vehicle speed. Usually, the balance gradient can be measured by the road sliding method in the field test [18].

The basic formulas adopted in the carbon emissions model are adapted from Yan and Xu [13], Gillespie [17], Crolla and Mashadi [18], Wong [19], Hucho and Sovran [27], and Rakha et al. [28], and have been widely verified with accuracy and reasonability.

#### Round trip of longitudinal slope

For a round trip of longitudinal segments, the uphill and downhill are symmetrical, besides both having the equal gradient and slope length. The greater slope, the less fuel consumption during the downhill travel of the truck and even the brake pedal needs to be depressed to control the stability of the truck speed; additionally, the truck would consume more fuel during the uphill travel.

When the gradient of the longitudinal slope is below the balance gradient, the truck requires the driving energy during downhill travel. The energy conversion correlation is demonstrated in Eqs (21)–(23). Compared to the energy output by the engine on the flat road section, the energy difference on the uphill and downhill sections is offset, as shown in Eqs (24)–(26).

$$W_{rt}=W_{up}+W_{down},$$ (21)

$$W_{up}=W_{a}+W_{r}+E_{g}+W_{t(up)},$$ (22)

$$W_{down}=W_{a}+W_{r}-E_{g}+W_{t(down)},$$ (23)

$$W_{flat}=2W_{a}+2W_{r}+2W_{t(flat)},$$ (24)

$$W_{t(up)}+W_{t(down)}=2W_{t(flat)},$$ (25)

$$W_{rt}=W_{flat},$$ (26)

where W_rt_ denotes the energy output by the engine (J) for the truck during the round trip of a longitudinal segments. W_up_ and W_down_ refers to the energy output by the engine (J) for the truck on uphill and downhill segments of symmetrical longitudinal slope combination road, respectively. W_flat_ denotes the energy output by the truck engine (J) on the flat straight road with equal mileage. W_t(flat)_, W_t(up)_, W_t(down)_ indicate the energy loss (J) in the transmission system on the flat road, uphill, and downhill segments, respectively.

When the gradient is equal to the balance gradient, the truck's energy conversion correlations on the round trip of the longitudinal slope is demonstrated in Eq (27). When predicting the dynamic performance of a truck, the difference between W_t_ and W_t_' is generally ignored [18]. It can be obtained that the energy output by the engine on the round-trip longitudinal slope section in this scenario is approximately equal to that on the flat road section, as shown in Eq (28).

$$W_{rt}=W_{up}=2W_{a}+2W_{r}+W_{t(up)}+W_{t}^{\prime },$$ (27)

$$W_{rt}=W_{up}\approx W_{flat},$$ (28)

In the round trip of longitudinal slope segments where the gradient is above the balance gradient, the energy conversion correlations of the truck is demonstrated in Eqs (29) and (30). The balance gradient determines whether the additional carbon emissions emitted by truck during uphill segments on the round-trip of the longitudinal slope.

$$W_{rt}=W_{up}=2W_{a}+2W_{r}+W_{t(up)}+W_{t}^{\prime }+W_{h},$$ (29)

$$W_{rt}=W_{up}\approx W_{flat}{+W}_{h},$$ (30)

### Field test

Field tests can directly reflect on the carbon emissions of trucks subjected to the actual conditions of different longitudinal slope segments. In the current research work, the fuel consumption test was carried out on the flat, uphill, and downhill segments to verify the reliability of the carbon emissions model of trucks on the flat road, uphill, downhill, and round-trip longitudinal slope segments. Moreover, the balance gradient test was carried out on the downhill segments.

The fuel consumption test requires the trucks to maintain a cruise speed on the test road segments. The balance gradient test was carried out on the downhill sections with different gradients. Aimed at obtaining the balance gradient of the common trucks on the highway, the trucks were required to slide downhill in a non-neutral position for attaining the cruise speed that trucks could maintain. In the field test, every group of data was collected and averaged at least 30 times on the longitudinal slope with a specified gradient.

#### Instruments and trucks

The diesel consumption instrument (Shenzhou JDSZ-EP-1-1D, Shanghai, China) was employed for the measurement of the trucks' fuel consumption, speed and travel time. The anemometer monitoring instrument (Xima AS8336, Guangdong, China) was employed for measuring the wind speed. Breeze with wind speed below 6.0m/s has almost no effect on the movement of ground objects. The wind speed was required not greater than 6.0m/s and allowed to fluctuate within the range of 2.0m/s [27]. Data from each group of tests that meet this requirement were considered valid. The average measured wind speed was taken as the wind speed value. An AxleLightRLU11 vehicle classification statistical instrument was placed on the test roads before the test for the selection for the trucks that have large traffic volumes. In accordance with the traffic volume data on the test road and the development prospects of trucks, three types of typical trucks were selected as the dominant vehicle types: one medium truck (Dongfeng Tianjin DFH5160CCYBX1JV), one heavy truck (FAW Jiefang CA5310CCYP66K2L7T4E5), and one tractor (FAW Jiefang A4250P66K24T3E5). The Dongfeng Tianjin (truck I) is equipped with a 4-cylinder ordinary compression-ignition diesel engine, coupled with 4.752L displacement, 5.64m^2^ frontal area, weighing 15 t at full load and 7 t at no load, two years age, and aerodynamic resistance coefficient of 0.49. The Jiefang heavy truck (truck II) is equipped with a 6-cylinder Xichai compression-ignition diesel engine, besides the 8.6L displacement, 5.64m^2^ frontal area, two years age, and aerodynamic resistance coefficient of 0.43. The masses at no load and full load are 13 t, and 28 t, respectively. The Jiefang tractor (truck III) is equipped with a 6-cylinder Xichai compression-ignition diesel engine, a displacement of 11.05L, a frontal area of 6.08m^2^, two years age, and an air resistance coefficient of 0.656. The no-load and full-load masses are 7.8 t and 40 t, respectively. The test trucks were supplied with 0# diesel during the field test. To ensure the reliability of the test data, there are ten trucks of each type of test truck were used for field testing.

#### Participants

Driver performance is different, which depends on the personal driving preferences and experience; accordingly, drivers were screened before the field test in a bid to make sure that they were not just sufficiently experienced but familiar with the road as well. Each driver was provided ten days of training and testing for the prevention of any incorrect driving operations from impacting the test results. The driver was required to maintain a cruise speed normally, together with avoiding any aggressive driving behavior, and maintaining a safe distance from the vehicle in front or side of them. The driving behavior of the test truck was not impacted by the other vehicles on the road. The lane change behavior and lane choice preference were not allowed [29]. 30 male drivers having between 15 and 20 years of driving experience passed the test. Each test truck type was assigned ten drivers. All participants gave verbal informed consent before participation in this study.

#### Test routes

The balance gradient tests and fuel consumption tests were carried out in the Xunyi-Qiupotou second class road (road I), the Hanzhong-Mianxian first class highway (road II), and the Xianyang-Xunyi expressway (road III). Test road sections selected from the road I was composed of the asphalt pavement type. The road surface is poor with a rolling resistance coefficient of 2.5. The pavement type of road II is asphalt pavement, whereas the road surface condition is fair. The rolling resistance coefficient is 1.5. The pavement type of road III is asphalt pavement. The road surface has an excellent condition. The rolling resistance coefficient is 1.25.

#### Other factors

The current research work primarily aimed at observing the correlation between carbon emissions and gradients, and the numbers of measures were assumed for preventing interference from factors like traffic flow conditions [7,30] and environmental conditions [31,32]. The test road sections were required to be the basic road section, and apart from the special sections [32], for instance, tunnels, bridges, interchanges, and toll stations. There is observed a free-flow pattern during the test. The weather was fine and remained basically fixed during the test.

## Results

**Fig 1:**
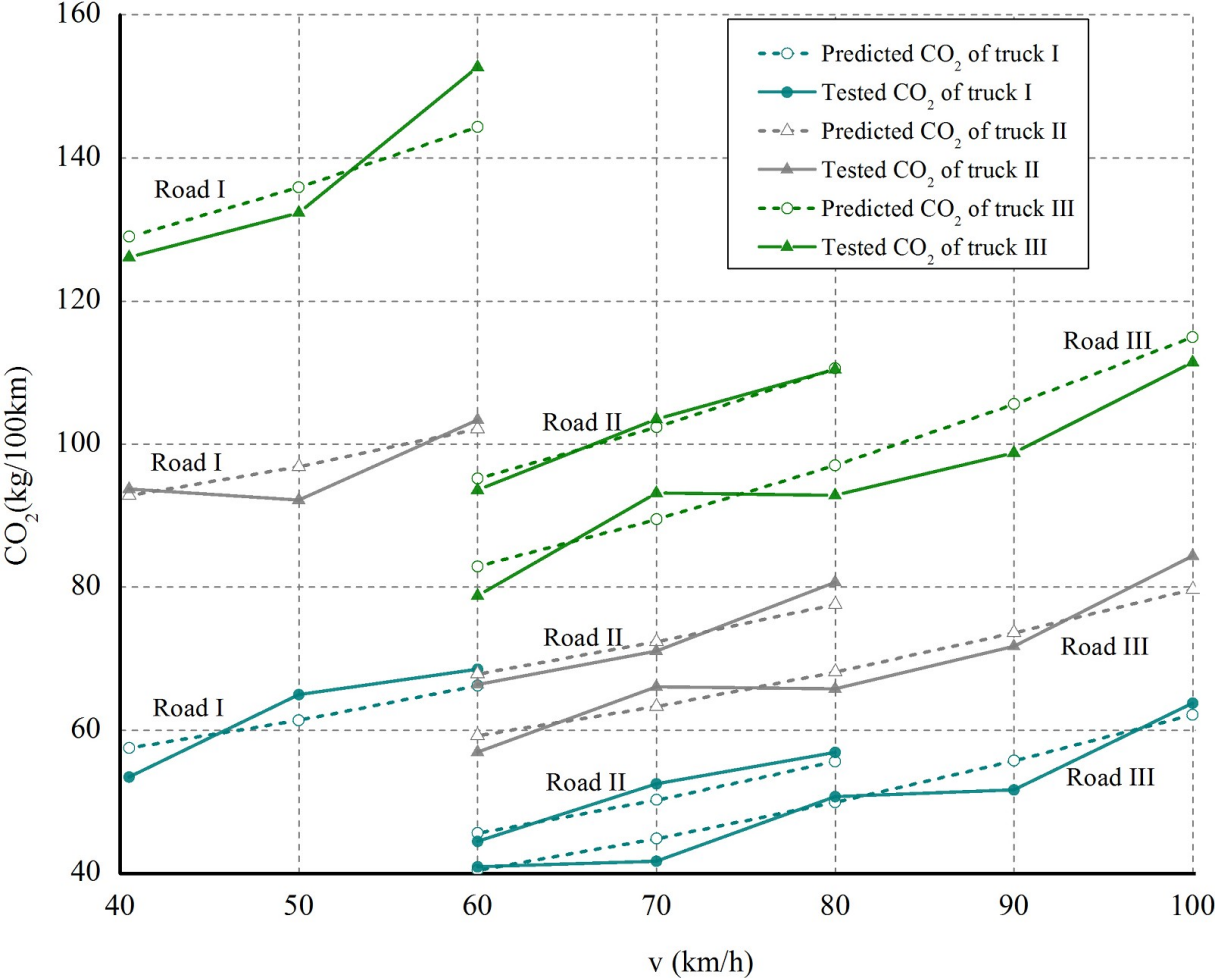
Trucks' CO_2_ emissions on flat road sections.

Fig 1 presents the carbon emissions of test trucks on the flat road sections of road I, road II, road III. It was considered the speed limit regulation on different road grades [33], thus test trucks were required to maintain cruising speed on the flat sections of road I, road II, road III, with a speed range of 40km/h to 60km/h, 60km/h to 80km/h, and 60km/h to 100km/h, respectively. The detailed description and test data are shown in S1 Table. The maximum relative errors of the measured and the predicted carbon emissions of test trucks is 7.87%, indicating the high accuracy of the proposed quantitative carbon emission model of diesel trucks on the flat road sections.

The measured and predicted balance gradients of trucks during downhill travel on various road grades are illustrated in Table 1. It indicates that the model of the downhill segments carries high reliability. It was considered that the different road grades and truck loads. There are different balance gradients for the trucks having different loads and on various road grades. The rule of balance gradient from the shallow to steep grades versus velocities is in line with the research results of Chang and Morlok [10].

**Table 1:** Balance gradient of trucks on downhill segments.

Road	v (km/h)	Truck I (no load)		Truck I		Truck II (no load)		Truck II		Truck III (no load)		Truck III	
		i_BT_ ^a^ (%)	i_BP_ ^b^ (%)	i_BT_ (%)	i_BP_ (%)	i_BT_ (%)	i_BP_ (%)	i_BT_ (%)	i_BP_ (%)	i_BT_ (%)	i_BP_ (%)	i_BT_ (%)	i_BP_ (%)
I	40	2.50	2.51	2.00	2.06	2.00	2.09	1.90	1.86	2.80	2.74	1.90	1.88
	50	3.00	2.90	2.30	2.29	2.30	2.33	2.00	2.03	3.30	3.20	2.00	2.05
	60	3.50	3.33	2.60	2.54	2.60	2.59	2.30	2.20	3.80	3.72	2.30	2.22
II	60	2.20	2.22	1.70	1.63	1.70	1.66	1.30	1.37	2.50	2.52	1.30	1.39
	70	2.50	2.61	1.90	1.84	1.90	1.88	1.50	1.50	3.00	3.00	1.50	1.53
	80	3.00	3.05	2.20	2.08	2.20	2.13	1.70	1.65	3.50	3.54	1.70	1.68
III	60	1.80	1.82	1.30	1.35	1.30	1.37	1.20	1.13	2.00	2.06	1.20	1.15
	70	2.00	2.17	1.50	1.53	1.50	1.57	1.30	1.25	2.50	2.49	1.30	1.27
	80	2.50	2.57	1.80	1.74	1.80	1.79	1.50	1.38	3.00	2.98	1.50	1.40
	90	3.00	3.00	2.00	1.97	2.00	2.03	1.60	1.51	3.50	3.52	1.60	1.55
	100	3.50	3.49	2.20	2.22	2.20	2.29	1.80	1.66	4.00	4.12	1.80	1.70

^a^ i_BT_ represents the tested balance gradient.

^b^ i_BP_ represents the predicted balance gradient.

Table 2 demonstrates the predicted and measured loaded truck's CO_2_ emissions on the flat, uphill, downhill, and round-trip longitudinal slope sections. Trucks were required to maintain a cruise speed on the road sections of road III (Nos. 1–20) and road II (Nos. 21–38). There is a small difference between the measured and predicted carbon emissions data, and the maximum relative errors of the test trucks is 9.99%. It suggests that the truck's CO_2_ emission model on uphill, downhill segments offers high accuracy. It highlights that, in cases II-IV, the truck's CO_2_ emissions on downhill segments are the ones, which are at the idle state.

**Table 2:** Truck's CO_2_ emissions on different longitudinal slope segments.

No.	v (km/h)	i (%)	Truck I					Truck II					Truck III					Diff.^a^ (%)	Diff. of Rule^b^ (%)
			Case	Predicted CO_2_ (kg/100 km)		Tested CO_2_ (kg/100 km)		Case	Predicted CO_2_ (kg/100 km)		Tested CO_2_ (kg/100 km)		Case	Predicted CO_2_ (kg/100 km)		Tested CO_2_ (kg/100 km)			
				Uphill	Downhill	Uphill	Downhill		Uphill	Downhill	Uphill	Downhill		Uphill	Downhill	Uphill	Downhill		
1	60	0.5	I	55.03	25.96	54.55	26.93	I	84.54	34.06	81.24	31.96	I	118.98	46.90	119.10	48.07	6.58	6.08
2	70	0.5	I	59.21	30.55	56.62	29.00	I	88.43	38.30	84.75	35.72	I	125.29	53.74	119.68	52.33	7.22	8.81
3	80	0.5	I	64.11	35.86	58.97	34.50	I	93.08	43.28	95.64	40.59	I	132.63	61.59	122.40	56.98	8.72	7.85
4	80	1.4	I	92.50	7.47	92.22	7.08	II	140.33	5.95	131.38	6.02	II	200.30	6.33	188.17	6.41	6.81	4.81
5	90	1.5	I	101.24	10.31	92.60	9.58	II	150.89	5.29	137.49	5.80	II	216.08	5.63	203.19	5.73	9.74	5.76
6	100	1.5	I	107.46	16.95	104.53	17.23	II	156.79	4.76	150.76	4.98	II	225.21	5.07	220.12	5.58	9.28	7.72
7	70	1.4	II	87.60	3.77	79.97	3.75	IV	135.68	6.80	140.80	6.74	IV	192.97	7.24	201.25	7.33	9.54	-
8	90	1.8	II	110.70	2.93	100.75	3.03	IV	166.64	5.29	167.16	5.40	IV	238.64	5.63	241.41	5.50	9.88	-
9	80	1.5	I	95.66	4.32	88.86	4.06	III	145.58	5.95	138.06	5.90	III	207.82	6.33	195.96	6.21	7.65	9.44
10	60	1.2	II	77.11	4.40	78.37	4.51	III	121.29	7.94	112.84	8.01	III	171.61	8.44	163.94	8.20	7.48	9.23
11	100	1.8	I	116.92	7.48	112.26	7.73	III	172.54	4.76	172.65	4.60	III	247.77	5.07	237.71	5.03	4.23	8.93
12	60	1.3	III	80.27	4.40	76.64	4.38	IV	126.54	7.94	135.73	8.19	IV	179.13	8.44	183.21	8.37	6.78	1.03
13	80	1.8	III	105.12	3.30	103.69	3.25	IV	161.33	5.95	147.13	5.90	IV	230.38	6.33	226.17	6.42	9.65	5.43
14	90	2	III	117.01	2.93	108.40	2.95	IV	177.14	5.29	173.17	5.28	IV	253.68	5.63	249.96	5.56	7.94	7.75
15	100	2.2	III	129.54	2.64	122.71	2.61	IV	193.54	4.76	188.93	4.65	IV	277.85	5.07	295.19	5.01	5.88	1.78
16	60	3	IV	133.89	4.40	125.86	4.43	IV	215.78	7.94	212.84	7.90	IV	306.97	8.44	305.95	8.10	6.38	-
17	70	3	IV	138.07	3.77	132.53	3.75	IV	219.68	6.80	218.25	6.92	IV	313.28	7.24	311.49	7.32	4.18	-
18	80	3	IV	142.97	3.30	136.82	3.30	IV	224.33	5.95	224.46	5.90	IV	320.62	6.33	319.18	6.56	4.50	-
19	40	1.5	I	101.25	16.50	100.79	15.98	I	168.63	19.24	164.51	17.52	I	237.24	24.14	225.95	22.80	9.81	9.21
20	50	1.5	I	103.86	21.58	105.95	23.12	I	171.65	24.28	166.60	25.57	I	242.56	32.58	221.49	29.84	9.51	5.04
21	60	1.5	I	107.47	27.66	106.05	26.08	I	175.93	30.59	168.89	32.54	I	249.44	42.59	241.03	42.99	6.05	7.00
22	40	1.8	I	110.72	7.04	110.29	6.68	II	184.38	11.90	169.63	11.95	II	259.80	12.66	244.92	12.71	8.70	9.40
23	60	2	II	123.24	11.89	121.93	12.06	II	202.18	7.94	200.65	8.01	II	287.04	8.44	280.54	8.59	2.32	5.33
24	40	1.9	II	113.87	6.59	110.12	6.82	III	189.63	11.90	179.13	11.81	III	267.32	12.66	264.95	12.16	5.86	9.87
25	60	2.3	II	132.71	4.40	125.82	4.56	III	217.93	7.94	208.41	8.03	III	309.60	8.44	297.69	8.99	6.04	4.85
26	40	2	III	117.03	6.59	110.45	6.60	IV	194.88	11.90	202.58	11.93	IV	274.84	12.66	300.32	11.72	8.49	9.47
27	50	2.3	III	129.09	5.27	131.37	5.25	IV	213.65	9.52	197.25	9.81	IV	302.71	10.13	290.03	10.31	8.31	5.13
28	60	2.6	III	142.17	4.40	132.17	4.52	IV	233.68	7.94	227.72	7.70	IV	332.16	8.44	330.58	8.47	7.57	0.25
29	40	3.5	IV	164.34	6.59	165.09	6.60	IV	273.63	11.90	271.68	11.84	IV	387.63	12.66	362.88	11.53	9.82	-
30	50	3.5	IV	166.95	5.27	165.58	5.25	IV	276.64	9.52	274.19	9.89	IV	392.95	10.13	380.78	10.98	7.74	-
31	60	3.5	IV	170.56	4.40	171.17	4.40	IV	280.92	7.94	279.10	7.72	IV	399.84	8.44	389.03	8.05	4.87	-
32	40	4	IV	180.11	6.59	173.80	6.50	IV	299.88	11.90	286.51	11.47	IV	425.23	12.66	407.77	11.68	8.42	-
33	50	4	IV	182.72	5.27	175.50	5.17	IV	302.89	9.52	292.69	9.60	IV	430.55	10.13	416.36	9.94	4.11	-

^a^ Diff. represents the difference between the measured and predicted carbon emissions data.

^b^ Diff. of Rule represents the difference of the rule, and the rule is that the carbon emissions of the trucks traveling on the round trip of a hilly route and flat straight route are basically equal.

As evident from the test results of the cases I-III, the average CO_2_ emissions on the round-trip longitudinal slope are basically equal to the CO_2_ emissions on the flat road. It is in line with the CO_2_ emission rules proposed above, with the maximum relative errors of 9.87%. In case IV, there is a large difference between the mean CO_2_ emissions on the round-trip longitudinal slope and the CO_2_ emissions on a flat road. The braking behavior usually takes place, causing the energy loss and lowering the total mechanical energy of the truck. As the law of conservation of energy puts forward, the energy loss was offset by a portion of propulsive energy as the truck goes uphill. In comparison with the flat road sections, there is the excess energy consumption and additionally carbon emissions generated on the uphill sections. It does not benefit the energy saving and emission reduction of the round-trip longitudinal segments.

The balance gradient segregates the carbon emission rules of trucks on the round-trip longitudinal slope segments into two types. The balance gradient refers to the minimum longitudinal gradient value, impacting the vehicle's CO_2_ emissions on the round-trip longitudinal slope, suggesting that there would be no difference in CO_2_ emissions among the different routes that have different road grade profiles within such a range.

The balance gradient correlates with the low carbon design of the vertical profile. With the gradient being larger in comparison with the balance gradient, the longer the slope length, and the greater the brake energy loss during downhill driving, the greater the truck propulsive energy supplied during uphill travel. This is not conducive to both energy-saving and CO_2_ emission reduction. On the road that has an integral subgrade, the gradient of the downhill could not be too large, and the excess energy supply of the uphill on the opposite road requires consideration. Considering the perspective of the avoidance of the excess energy consumed on uphill and the braking energy loss during the downhill, as compared with the energy on the flat road, it is indicated that, concerning the highways having the integral subgrade, the gradient should be avoided to be above the balance gradient.

## Discussion

Based on the law of conservation of mechanical energy, the law of the first law of thermodynamics and the vehicle longitudinal dynamics theory, the analysis of the energy conversion during several operating conditions on the uphill, downhill, and the round-trip of the longitudinal slope were primarily performed. The CO_2_ emissions model of diesel trucks on these segments were put forward as well, together with clarifying the balance gradient of the different highway grades. The impact of the gradient on energy saving and CO_2_ emission reduction is demonstrated as well.

The influence of the longitudinal slope design indicator on diesel trucks' carbon emissions was assessed in the present study. Especially, from the perspective of the travel on the round-trip of a hilly route, when the gradient exceeds the balance gradient, the energy loss caused by braking during downhill travel was nullified by the extra energy supplied on uphill roads, thereby causing an increase in fuel consumption while driving on the round trip of a hilly route compared to the flat straight route. The balance gradient refers to the minimum longitudinal gradient value, which leaves an impact on the CO_2_ emissions of the truck on the round-trip longitudinal slope. When the slope is not greater than the balance gradient, the carbon emissions of vehicles traveling on the round trip of a hilly route and flat straight route are basically equal.

The results of the current investigation can be applied to the typical trucks maintaining a cruising speed on Chinese highways. The truck speed was idealized as a uniform speed in this model to account for the effects of gradient on the carbon emissions of diesel trucks, which leaves the proposed model has a limitation to predict the carbon emissions of diesel trucks that maintained a fluctuate speed during travel. It should be considered in future studies to more accurately estimate the carbon emissions of truck in real-world driving conditions. Additionally, three common diesel trucks were selected as typical truck types in the model proposed. Therefore, they do not represent the carbon emissions of all vehicles passing by on the expressway. The carbon emission quantification model of the typical diesel trucks in China were the focus of this study. Models for other types of vehicles in other regions may be modified to account for multiple factors, such as the different road conditions (pavement type, and pavement evenness condition), the vehicle characteristics (vehicle performance, engine type, speed, frontal area, vehicle load, tire type, fuel type) and the climate of the region.

## Conclusions

The carbon emission quantification model, presented in the current paper can provide support for data and a viable reference not only for the low-carbon road design but also for the low-carbon route selection. The results of this study will help to evaluate green and environmentally sustainable highways. The proposed model is also suitable for the forecast of diesel fuel consumption based on a strong correlation between fuel consumption and CO_2_ emissions.

The clarification of the balance gradient is very significant when it comes to the design of the low-carbon highway and has the potential to provide a reference to the design of the vertical road profile. In view of the fact that the road subgrade is usually an integral subgrade, the gradient of the highway should be below the balance gradient, primarily aimed at achieving a low-carbon operation of the two-way traffic flow.

## Supporting information

S1 Table Trucks' CO_2_ emissions on flat road sections. (DOCX)
