# Supplementary material for: Modelling carbon emissions of diesel trucks on longitudinal slope sections in China
Source: PLoS One. 2020 Jun 16;15(6):e0234789. doi: 10.1371/journal.pone.0234789 (PMC7297377; doi:10.1371/journal.pone.0234789)
Supplement: S1 Table — (DOCX) [file pone.0234789.s001.docx]

**S1 Table. Trucks' CO_2_ emissions on flat road sections.**

| Truck type | Road | Road grade | Average speed (km/h) | Driving distance (km) | Predicted CO_2_  (kg/100 km) | Tested CO_2_ (kg/100 km) |
| --- | --- | --- | --- | --- | --- | --- |
| Truck I | Road I | Second grade highway | 40 | 4 | 57.54 | 53.46 |
|  |  |  | 50 | 4 | 61.38 | 64.98 |
|  |  |  | 60 | 4 | 66.23 | 68.52 |
|  | Road II | First class highway | 60 | 7 | 45.61 | 44.46 |
|  |  |  | 70 | 7 | 50.25 | 52.52 |
|  |  |  | 80 | 7 | 55.62 | 56.89 |
|  | Road III | Expressway | 60 | 10 | 40.46 | 40.93 |
|  |  |  | 70 | 10 | 44.84 | 41.68 |
|  |  |  | 80 | 10 | 49.95 | 50.72 |
|  |  |  | 90 | 12 | 55.74 | 51.67 |
|  |  |  | 100 | 12 | 62.17 | 63.79 |
| Truck II | Road I | Second grade highway | 40 | 4 | 92.84 | 93.73 |
|  |  |  | 50 | 4 | 96.87 | 92.16 |
|  |  |  | 60 | 4 | 102.16 | 103.37 |
|  | Road II | First class highway | 60 | 7 | 67.85 | 66.40 |
|  |  |  | 70 | 7 | 72.34 | 71.06 |
|  |  |  | 80 | 7 | 77.59 | 80.68 |
|  | Road III | Expressway | 60 | 10 | 59.27 | 56.94 |
|  |  |  | 70 | 10 | 63.34 | 66.05 |
|  |  |  | 80 | 10 | 68.15 | 65.77 |
|  |  |  | 90 | 12 | 73.63 | 71.73 |
|  |  |  | 100 | 12 | 79.71 | 84.38 |
| Truck III | Road I | Second grade highway | 40 | 4 | 129.00 | 126.11 |
|  |  |  | 50 | 4 | 135.88 | 132.34 |
|  |  |  | 60 | 4 | 144.33 | 152.69 |
|  | Road II | First class highway | 60 | 7 | 95.18 | 93.58 |
|  |  |  | 70 | 7 | 102.37 | 103.49 |
|  |  |  | 80 | 7 | 110.58 | 110.43 |
|  | Road III | Expressway | 60 | 10 | 82.89 | 78.79 |
|  |  |  | 70 | 10 | 89.47 | 93.16 |
|  |  |  | 80 | 10 | 97.06 | 92.83 |
|  |  |  | 90 | 12 | 105.58 | 98.77 |
|  |  |  | 100 | 12 | 114.97 | 111.42 |
